# Studies on Transition Metal-Quercetin Complexes Using Electrospray Ionization Tandem Mass Spectrometry 

**DOI:** 10.3390/molecules20058583

**Published:** 2015-05-13

**Authors:** Yuanzhen Liu, Mingquan Guo

**Affiliations:** 1 Key Laboratory of Plant Germplasm Enhancement and Specialty Agriculture, Wuhan Botanical Garden of Chinese Academy of Sciences, Wuhan 430074, China; E-Mail: lyz19851001@wbgcas.cn; 2 The Keck School of Medicine, University of Southern California, Los Angeles, CA 90089, USA

**Keywords:** flavonoid, ESI-MS^n^, metal complexes, CID, fragmentation mechanism

## Abstract

To systematically study the effects of the number of *d* electrons of the first transition metal ions (Fe, Co, Ni, Cu and Zn) on the formation and stability of metal flavonoid complexes, we took the quercetin/M^2+^ complex as a model system to investigate the structures and properties of these complexes. Based on considerable structural information obtained through ESI-MS^n^, all of the first transition metal ions (Fe^2+^, Co^2+^, Ni^2+^, Cu^2+^ and Zn^2+^) were found to form different complexes with quercetin, while with the number of chelating flavonoids decreasing along with the reduction of the metal ionic radius. Quercetin forms different complexes with the above metal divalent ions through its 5-OH and 4-carbonyl groups; the complex stability is highly dependent on both the metallic ion and the flavonoid chelator itself. As for the central ion (M^2+^), when chelated with quercetin to form the complex, the stability of the complex decreased in the following order: Cu^2+^ > Ni^2+^ > Co^2+^ > Fe^2+^ > Zn^2+^. With flavonoid: metal stoichiometries at 2:1, the complexes formed between quercetin and metal ions (Fe^2+^, Ni^2+^, Co^2+^ and Zn^2+^) have the similar fragmentation mechanism, while Cu^2+^ displayed different fragmentation mechanism due to the concurrent oxidation.

## 1. Introduction

Flavonoids are a class of widely spread plant secondary metabolites that have shown promising anti-oxidant, anti-virus, anti-inflammatory and anti-cancer activities [1,2]. In general, the anti-oxidant mechanisms of flavone compounds are mainly due to their ability to scavenge free radicals. Another mechanism may result from the interactions between flavonoids and transition metal ions to form complexes that prevent the participation of metal ions in free radical generating processes, thus exhibiting an anti-oxidant behavior [3]. Recent studies indicated that metal-flavonoid complexes have good biological and pharmacological activities, and some of which have been successfully used in clinical practices [4,5]. Meanwhile, the flavonoids, as natural metal chelators, have also played significant roles in metal bio-utilization and reduction of heavy metal toxicity, and may suppress the Fenton response and lipid peroxidation [6]. The coordination of flavonoids with Cu^2+^, Mn^2+^, or Fe^2+^ metallic ions, may simulate the catalyzed center of the hyperoxide dismutase in the metal coordinate structure, thus displayed the superoxide dismutase (SOD) activity [7]. Therefore, research on the metal flavonoid complexes is very helpful in developing new medicines, based on these complexes and exploring new ways in screening, discovery and development of new drugs.

Quercetin (3,3',4',5,7-pentahydroxyflavone) is one of the most bioactive and common dietary flavonoids, which widely exists in the flowers, leaves, and fruits of many plants. However, due to its poor solubility, quercetin was found to be difficult to be absorbed into the body, thus resulted in poor bioavailability *in vivo*. Fortunately, it has been reported that quercetin can form complexes with transition metal ions, such as Cu^2+^, Mn^2+^ and Fe^2+^. These quercetin-metal complexes exhibit broad biological activities with increasing bioavailability, such as anti-oxidation, anti-bacterial, anti-tumor, and the ability to affect many kinds of enzymatic activities [8,9,10,11]. Not only can these quercetin/metallic ion complexes enhance its bioavailability and change the way to deliver quercetin *in vivo*, but they also promote new pharmacological activity (e.g., SOD activity) [12,13,14]. Although the traditional methods (e.g., UV/Vis and IR absorption spectroscopy, Raman spectra) have been used to elucidate the structures of metal flavonoid complexes in numerous studies, they can only give indirect evidence for flavonoid-transition metal complexation [15,16,17,18,19,20,21,22]. Recently, electrospray ionization tandem mass spectrometry (ESI-MS) has become a powerful tool in the analysis of metal-flavonoid complexes [23,24], which could provide abundant fragment information for the characterization of those complex labile structures, such as the most important information regarding the stoichiometry and chelating sites of the metallic complexes [25,26,27]. By using soft ionization mass spectrometry, the first systematic investigation on the complexes between quercetin and five kinds of first transition metal ions (Fe^2+^, Co^2+^, Ni^2+^, Cu^2+^ and Zn^2+^) was conducted in this study, and showcased the power and potential wide applicability of this strategy for the in-depth characterization of metallic complexes.

## 2. Results and Discussion

**Scheme 1:**
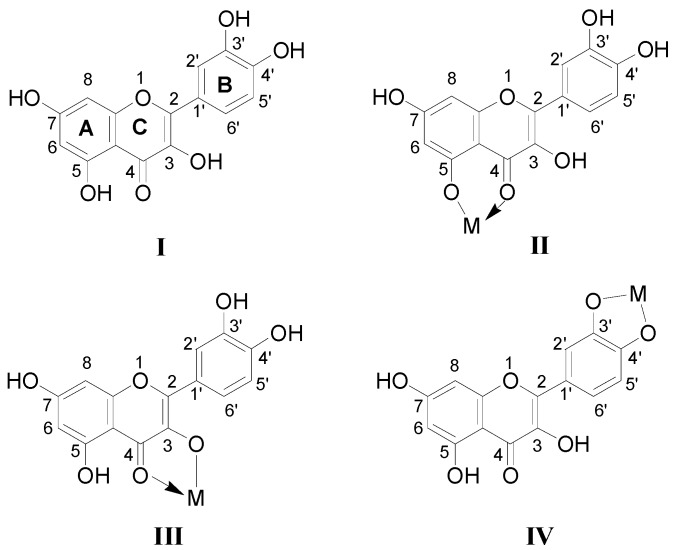
Structures of Quercetin and its proposed chelating complexes with M^2+^.

To systematically study the effects of the number of *d* electrons of the first transition metals (Fe, Co, Ni, Cu and Zn) on the formation and stability of metal flavonoid complexes, quercetin/M^2+^ complexes were chosen as a model system to investigate the structure and properties of these metallic complexes. Although the formation of transition metal-flavonoid complexes is quite complicated, ESI-MS^n^ can be used to detect the formation of metal-flavonoid complexes in solution with high sensitivity. The chemical structures of quercetin can be found in Scheme 1 (“Q” denotes quercetin hereafter). The positive scan mode was used throughout the mass spectrometric experiments. Collision-induced dissociation (CID) spectra were obtained by selecting a given complex with the stoichiometry of 2:1 in the first quadrupole and transferring it into the collision cell with argon. In order to optimize the conditions, the pressures and the voltages applied to the collision cell were tuned in the ranges of (1–4) × 10^−3^ mBar and 20–60 eV, respectively.

### 2.1. Full-Scan ESI-MS Analysis of M^2+^/Quercetin Complexes

**Figure 1:**
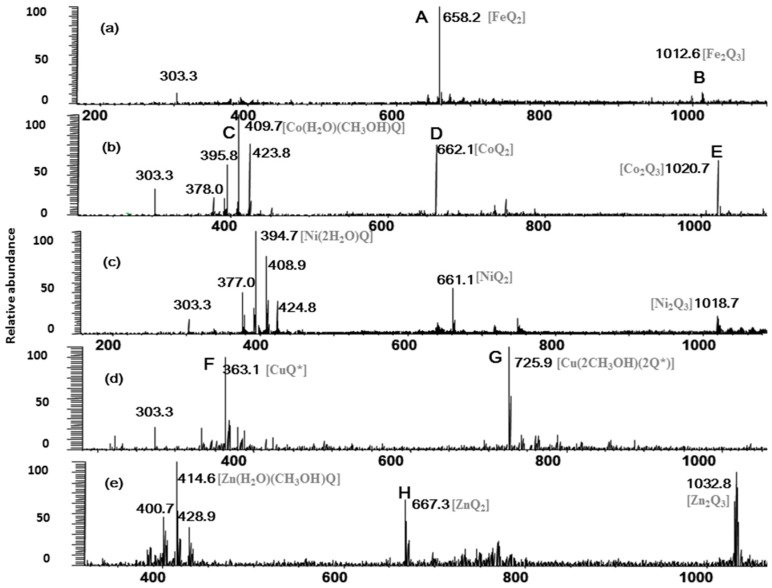
Full scan mass spectrum of the quercetin/M^2+^ complexes (Q*-oxidized quercetin). (a)–(e) for quercetin-Fe, -Co, -Ni, -Cu, and -Zn complexes, respectively.

The full-scan mass spectra of quercetin-metal complex were shown in Figure 1. Two quercetin-Fe complexes are observed in Figure 1a, where peak A was shown at *m/z* 658 and peak B at *m/z* 1012. According to their molecular weights, it was deduced that complex A corresponded to [(2Q-H)Fe]^+^ (molar ratio of Q/Fe = 2:1), and complex B was in agreement with [(3Q-3H)Fe_2_]^+^ (molar ratio of Q/Fe = 3:2). Similarly, peak D (*m/z* 662) and E (*m/z* 1020) in Figure 1b were corresponding to the quercetin-Co complexes [(2Q-H)Co]^+^ (molar ratio of Q/Co = 2:1) and [(3Q-3H)Co_2_]^+^ (molar ratio of Q/Co = 3:2), and the quercetin-Co complex also showed methanol and water adducts (peak C) at *m/z* 409 ([Co(H_2_O)(CH_3_OH)Q]^+^). In Figure 1d, redox reaction through the change of oxidation state of Cu was observed in our experiment, in which peaks F and G represented the quercetin-Cu complexes [Cu(Q*)]^+^ (molar ratio of Q/Cu = 1:1) and [Cu(2CH_3_OH)(2Q*)]^+^ (molar ratio of Q/Cu = 2:1), respectively (Q* denotes oxidized quercetin). The peak at *m/z* 303, corresponding to protonated quercetin ([Q+H]^+^) was found in all three positive full-scan mass spectra (Figure 1a–c). In addition, most of the other related peaks were also inferred and labeled in Figure 1. However, the chloride adducts have not been observed in the positive ion mode under our experimental conditions, and those adducts may be more easily observed in the negative ion mode.

From the full-scan mass spectra, several conclusions could be drawn. First, Fe^2+^ and quercetin mainly formed metal-flavonoid complexes with stoichiometry of 1:2 and a few complexes with stoichiometry of 2:3, while Cu^2+^ and quercetin formed complexes with stoichiometries 1:1 and 1:2. Secondly, Co^2+^, Ni^2+^, Zn^2+^ and quercetin formed metal-flavonoid complexes with stoichiometries of 1:1, 1:2 and 2:3. This indicated that Co^2+^, Ni^2+^, Zn^2+^ were easier to form metal-flavonoid complexes with higher stoichiometries than those of Cu^2+^, and they are in the following order: Fe^2+^ > Zn^2+^ > Co^2+^ > Ni^2+^ > Cu^2+^. We thus conclude that the first transition metallic ions (II) including Fe, Co, Ni, Cu and Zn can all form the different complexes with quercetin, and the number of chelating flavonoids decreases along with reduction of the five kinds of the first transition metal ionic radius. The reported metal ionic radius, number of *d* electrons, crystal field stabilization energies (CFSE) and other parameters [28] were shown in Table 1.

**Table 1:** The ionic radius, number of *d* electrons and CFSE of the first transition metals.

Metal Ion	Number of *d-*electrons	Metal *R.*	Space Configuration	CFSE(Dq)
Fe^2+^	d^6^	75 pm	Octahedron	−4
Co^2+^	d^7^	72 pm	Octahedron	−8
Ni^2+^	d^8^	70 pm	Octahedron	−12
Cu^2+^	d^9^	69 pm	Plane square	−12.3
Zn^2+^	d^10^	74 pm	Tetrahedron	0

### 2.2. Multistage Tandem Mass Spectrometry Analysis of M^2+^/Quercetin Complexes

In order to obtain more structural information of M^2+^/quercetin complexes and their relative stability, multistage tandem mass spectrometry was used to characterize the M^2+^/quercetin (molar ratio of Q/M = 2:1) complexes. The resulting spectra can provide important structural information regarding these complexes, which are very useful to further explore the dissociation mechanism and stabilities of the quercetin-metal complexes.

**Figure 2:**
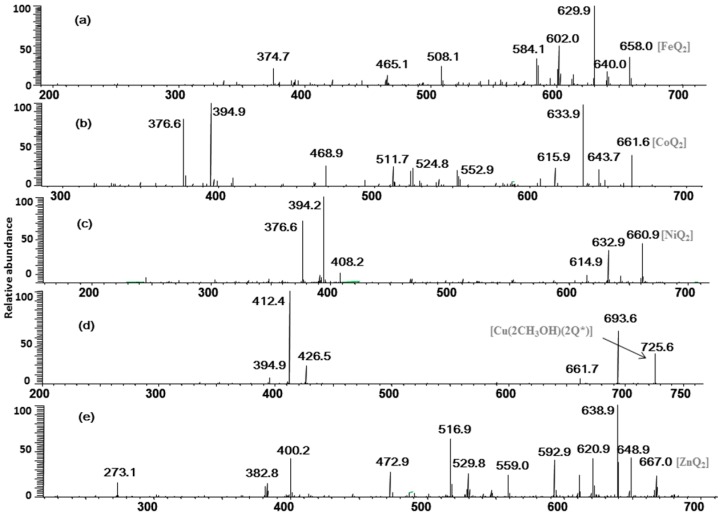
MS/MS spectrum of the quercetin/M^2+^ complexes (Q/M^2+^ = 2:1). (a)–(e) for quercetin-Fe, -Co, -Ni, -Cu, and -Zn complexes, respectively.

As shown in Figure 2a, the CID spectrum of the quercetin-Fe complex A (*m/z* 658, molar ratio of Q/Fe = 2:1), Figure 2a produced a lot of information with the generation of considerable product ions by a series of neutral losses, such as losses of CO and the H_2_O, as well as cleavages of the C-ring and chelating bonds.

These product ions were extremely important in inferring the chelating positions of the M^2+^/quercetin complexes. Eight ions at *m/z* 640, 630, 612, 602, 584, 508, 465, and 375 were present in the MS^2^ spectrum: the ion at *m/z* 640 was derived from the neutral loss of H_2_O (−18 Da); the most intensive product ion (*m/z* 630) came from the neutral loss of CO (–28 Da); The peaks at *m/z* 612, 602 and 584 were corresponding to the ions [A–H_2_O–CO]^+^, [A–2CO]^+^ and [A–2CO–H_2_O]^+^, respectively. The cleavage of C ring (retro Diels-Alder (RDA) reaction) of quercetin yielded the ion at *m/z* 508 by a neutral loss of 150 Da from the parent ion at *m/z* 658, and the *m/z* 508 ion was prone to the loss of one CO_2_ to produce the ion at *m/z* 465.

**Scheme 2:**
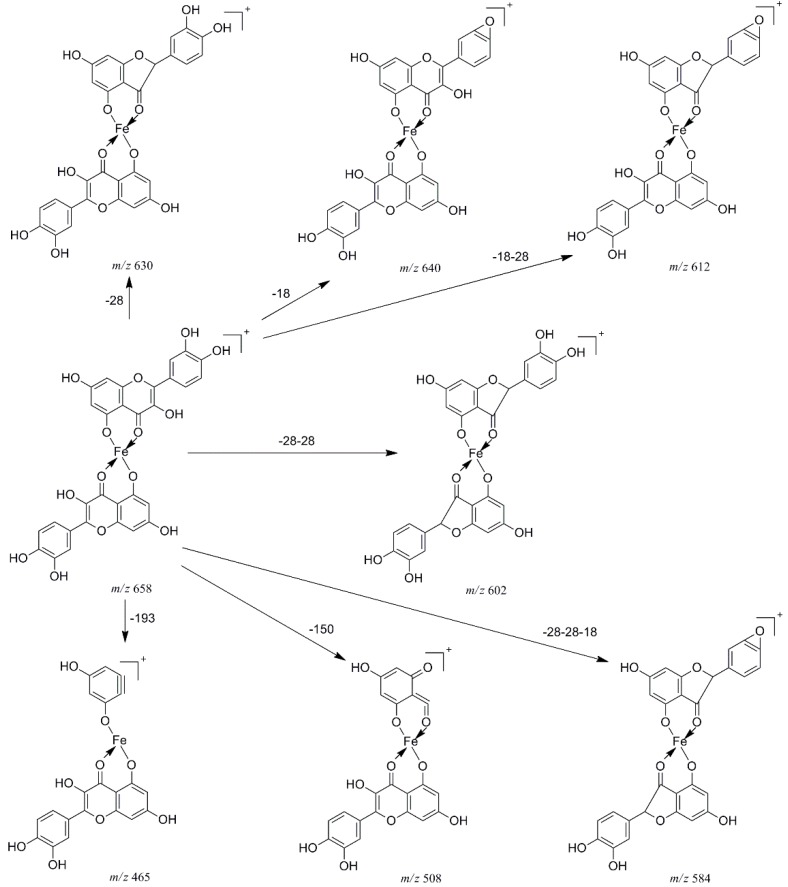
Proposed fragmentation mechanism of the quercetin-Fe complex A (*m/z* 658).

In the pathway of losing a quercetin aglycone (–302 Da), the *m/z* 375 ion is produced. Besides, it is reported that there are three possible complexation sites between metal ions and quercetin [29,30] (Scheme 1). The net charge (*q*) and charge density edge (Q and QR) of the primary coordination atoms [29,30], which are very important in determining the complexation sites, are summarized in Table 2. From the above results, it can be deduced that quercetin formed different complexes by its 5-OH and 4-carbonyl with these metal divalent ions, which is the most favorable fragmentation pathway, because it does not involve the cleavage of the chelating bonds in the RDA reaction. These results further indicated that the above metal divalent ion can form stable complexes in solution. For example, if complex A were just a simple adduct of the Fe^2+^ and quercetin (e.g., 1:2 complexes), some simple decomposition product ions should be observed. Based on the above discussion, the fragmentation mechanism of the quercetin-Fe complex A was proposed in Scheme 2.

**Table 2:** Charge density and net charge density of primary coordination atoms in flavonoids.

Atom	Net Charge (*q*)	Charge Density Edge (Q)	Energy-Weighted Charge Density Edge (QR)
O-3	−0.3712	0.0709	−0.1814
O-5	−0.3948	0.0018	−0.0046
O-3'	−0.3708	0.0422	−0.1081
O-4'	−0.3705	0.0169	−0.0432

The fragmentation pathways of quercetin-Co, -Ni, and -Zn complexes (*m/z* 662, 661 and 667) are quite similar. In Figure 2b–e for example, we took the CID spectrum of the quercetin-Zn complex H (*m/z* 667, molar ratio of Q/Zn = 2:1, Figure 1e to explain their dissociation mechanism and infer their complexation sites.

**Scheme 3:**
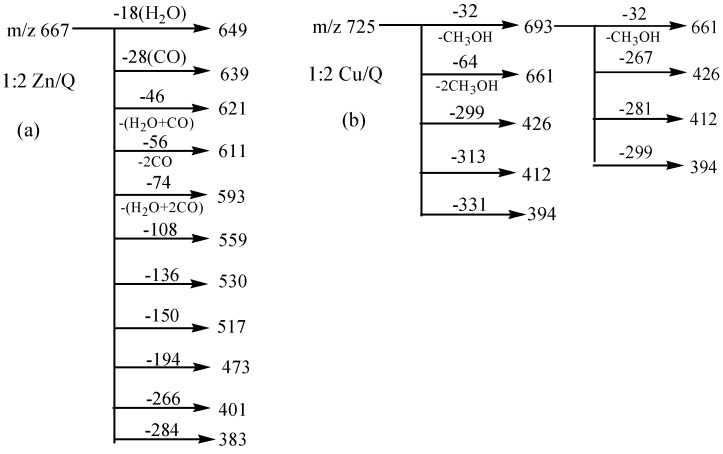
MS/MS Data analysis for 1:2 Zn/Q (**a**) and Cu/Q complexes (**b**).

The MS/MS spectrum of the quercetin-Zn complex H (*m/z* 667, molar ratio of Q/Zn = 2:1), in Figure 2e also yielded abundant product ions, including a series of ions with neutral losses, such as losses of CO and the H_2_O, as well as cleavages of the C-ring and chelating bonds. Almost all types of neutral losses of quercetin-Fe complex A was reproduced in the MS^2^ spectrum of the complex H, and the CID data of which was summarized in Scheme 3a. However, Zn^2+^ has greater polarizability than that of Fe^2+^ because it contains more *d-*electrons, and the quercetin-Zn complex produced more product ions than that of the quercetin-Fe complex. For instance, the peak at *m/z* 559 was produced by the loss of B ring of quercetin from *m/z* 667, and the ion at *m/z* 531 came from a further loss of CO from *m/z* 559; the other product ions, *m/z* 401 and *m/z* 274, arose from a loss of 266 Da and 394 Da from *m/z* 667, respectively. The CID spectra of quercetin-Co and quercetin-Ni complexes were almost identical with that of quercetin-Zn complex, which were shown in Figure 2b,c, respectively.

**Scheme 4:**
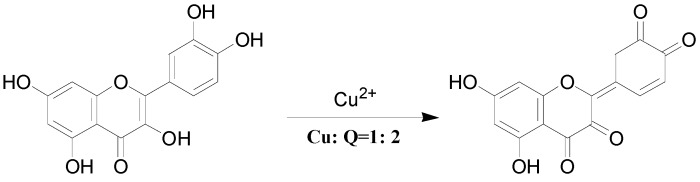
Oxidized structure proposed for quercetin by Cu^2+^.

Interestingly, Cu^2+^ displayed quite different dissociation mechanism as compared with other divalent ions above (Ni^2+^, Co^2+^, Fe^2+^ and Zn^2+^). This is partially because that Cu^2+^ has stronger oxidative activity, and quercetin is easier to be oxidized under acidic conditions. The possible oxidization sites of quercetin when oxidized by Cu^2+^ were reported as shown in Scheme 4 [23]. Obviously, quercetin could only form complexes with Cu^2+^ through 5-OH and 4-C=O once it was oxidized. In the CID spectrum of the quercetin-Cu complex G (*m/z* 725, molar ratio of Q/Cu = 2:1), shown in Figure 2d, five major product ions (at *m/z* 693, 661, 426, 412 and 394) were observed. The parent ions *m/z* 725 lost one and two CH_3_OH molecules to produce the *m/z* 693 and 661 ions, respectively (Scheme 3b). In fact, small molecules (H_2_O, CH_3_OH, *etc*.) adducts of metal complexes were usually found in the mass spectrometry [31].

**Figure 3:**
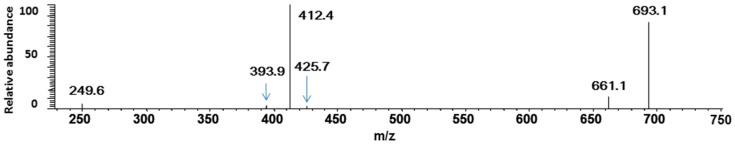
MS^3^ spectrum of Cu^2+^/Q complex M (725 > 693 >).

According to the masses of most fragments, we proposed that the ion at *m/z* 426 was derived from the neutral loss of an oxidized quercetin aglycone (−299 Da); the product ion at *m/z* 412 with the highest abundance was due to the neutral loss of one oxidized quercetin aglycone and the -CH_2_ groups of methanol (−313 Da = (−299 Da) + (−14 Da)); the ion at *m/z* 394 corresponded to the neutral loss of an oxidized quercetin aglycone and the adduct methanol molecule (−331 Da = (−299 Da) + (−32 Da)) (Scheme 3b). These inferences further suggested that three hydroxyl groups in quercetin had been oxidized in this process: quercetin was first oxidized by Cu^2+^ to produce the quercetin stable oxide, then it formed a 2:1 complex with Cu^2+^ through 5-OH and 4-C=O, and two methanol molecules were added to the quercetin-Cu complex. To investigate the above speculation, we performed an additional MS^3^ experiment on the *m/z* 693 ion. The resulting spectrum is shown in Figure 3. In this spectrum, four stable product ions at *m/z* 661, 425, 412, and 394 were observed.

In summary, our studies showed that quercetin was able to form a series of metal-flavonoid complexes through its 5-OH and 4-carbonyl groups with all of the first transition divalent metal ions (Fe^2+^, Co^2+^, Ni^2+^, Cu^2+^ and Zn^2+^). The stabilities and chelating sites of these metallic complexes were highly dependent on the properties of both the metallic ions and the flavonoid chelator, and could be successfully characterized by means of ESI-MS^n^.

## 3. Experimental Section 

### 3.1. Reagents and Chemicals

Standard quercetin was purchased from National Institutes for Food and Drug Control (Beijing, China). All the salts used in this study (FeCl_2_∙4H_2_O, CoCl_2_∙6H_2_O, NiCl_2_∙6H_2_O, CuCl_2_∙2H_2_O and ZnCl_2_) as well as the solvents (MeOH) were of analytical purity without further purification.

### 3.2. Sample Preparation

The quercetin standard and five transition metal chlorides (FeCl_2_∙4H_2_O, CoCl_2_∙6H_2_O, NiCl_2_∙6H_2_O, CuCl_2_∙2H_2_O and ZnCl_2_) were dissolved in methanol with a final concentration of 10 μM. Methanol solution of quercetin was mixed with five kinds of transition metal salt solutions with equal volume in the test tubes, respectively. Then the tubes were sealed. After 30 min ultrasonic treatment, the solution was subjected to mass spectrometry analysis.

### 3.3. Mass Spectrometry

All of the mass spectrometric experiments were performed on a Finnigan MAT LCQ^TM^ mass spectrometer (Finnigan, San Jose, CA, USA). Before each experiment, the instrument was calibrated using standard chemicals (caffeine, peptide MFRA and Ultramark 1621), and tuned following protocols provided by the instrument manufacturer. The scan range was from *m/z* 50 to 2000 with unit mass resolution, and the infusion rate was 3 μL/min. The spray voltage was set to 4.8 kV, and the capillary temperature was 180 °C. In the MS^n^ experiments, helium was used as collision gas, and the mass isolation width was 2.0 Da.

## 4. Conclusions

Metal flavonoid complexes are a promising class of natural drug leads, and the associated studies have attracted more and more attention. However, the systematic basic research of metal-flavonoid complexes is lagging behind, partly because very few efforts have been made to establish a set of activity screening and subsequent evaluation system for the comprehensive investigations on the structure and activity relationship of metal-flavonoid complexes. 

The effects of the number of *d*-electrons of the first transition metals on the formation and stability of metal-flavonoid complexes were systematically investigated in the present study. Although the formation of flavonoid-transition metal complexes is quite complicated, our results showed that ESI-MS^n^ can be used to study these complexes with several obvious advantages. First, the complexes can be detected in solution with high sensitivity. Not only can this technique provide direct evidence for the formation of the complexes in the solution, but can also be easily used to explore the information on the composition and stoichiometry of the complexes based upon the masses of complex ions and fragmentation ions of the complexes of interest. Second, the relative stabilities of complexes may be determined by means of the relative CID energies under the same experimental condition, which is very important in establishing a fast screening system for metal-flavonoid complexes. More importantly, ESI-MS^n^ can facilitate the determination of the complexation sites of the complexes, even of other side reactions (e.g., oxidization reaction) during the complexation process, thus provided a highly sensitive and fast screening method in exploring the structure and property of these interesting complexes. 

Using ESI-MS^n^ to characterize the metal-flavonoid complexes, we found that all of the first transition metallic ions (Fe^2+^, Co^2+^, Ni^2+^, Cu^2+^ and Zn^2+^) can form complexes with quercetin; the number of chelating flavonoids decreases along with the reduction of the metal ionic radius. Quercetin forms different complexes with the above metal divalent ion through its 5-OH and 4-carbonyl groups; the complex stability is highly dependent on both the metallic ion and the flavonoid chelator. As for the central ion (M^2+^), when chelated with quercetin to form the complex, it has the following order in stability: Cu^2+^ > Ni^2+^ > Co^2+^ > Fe^2+^ > Zn^2+^. With stoichiometry of flavonoid: metal at 2:1, the complexes formed between quercetin and metal ions (Fe^2+^, Ni^2+^, Co^2+^ and Zn^2+^) have similar dissociation mechanisms. However, Cu^2+^ displayed a different dissociation mechanism, mainly because of its relatively stronger oxidation capacity, and ESI-MS^n^ can readily examine this oxidation process. Thus, our study could be easily expanded to the analysis of other types of metal-flavonoid complexes, even other metallic complexes, and may greatly contribute to a better understanding of the chelating chemistry of flavonoids with transition metal ions and beyond, and further promote the research in the potential drug discovery and development from flavonoid metal complexes.
